# Modulation of intercolumnar synchronization by endogenous electric fields in cerebral cortex

**DOI:** 10.1126/sciadv.abc7772

**Published:** 2021-03-03

**Authors:** Beatriz Rebollo, Bartosz Telenczuk, Alvaro Navarro-Guzman, Alain Destexhe, Maria V. Sanchez-Vives

**Affiliations:** 1 Institut d’Investigacions Biomèdiques August Pi i Sunyer (IDIBAPS), Barcelona, Spain.; 2 Université Paris-Saclay, Centre National de la Recherche Scientifique (CNRS), Institut des Neurosciences, Gif sur Yvette, France.; 3 ICREA, Barcelona, Spain.

## Abstract

Neurons synaptically interacting in a conductive medium generate extracellular endogenous electric fields (EFs) that reciprocally affect membrane potential. Exogenous EFs modulate neuronal activity, and their clinical applications are being profusely explored. However, whether endogenous EFs contribute to network synchronization remains unclear. We analyzed spontaneously generated slow-wave activity in the cerebral cortex network in vitro, which allowed us to distinguish synaptic from nonsynaptic mechanisms of activity propagation and synchronization. Slow oscillations generated EFs that propagated independently of synaptic transmission. We demonstrate that cortical oscillations modulate spontaneous rhythmic activity of neighboring synaptically disconnected cortical columns if layers are aligned. We provide experimental evidence that these EF-mediated effects are compatible with electric dipoles. With a model of interacting dipoles, we reproduce the experimental measurements and predict that endogenous EF–mediated synchronizing effects should be relevant in the brain. Thus, experiments and models suggest that electric-dipole interactions contribute to synchronization of neighboring cortical columns.

## Teaser

Endogenous electric fields modulate synchronization across cerebral cortex columns compatible with electric-dipole interactions.

## INTRODUCTION

The cerebral cortex is organized into circuits of strongly interconnected neurons in a conductive medium. During deep sleep, neuronal connectivity and neuronal properties interact to generate recurrent synchronized synaptic activity leading to periods of activity (Up states) interspersed with silent periods (Down states). This stereotypical pattern is manifested as slow oscillations, a ≤1-Hz rhythm that dominates the cortical network during slow-wave sleep (*1*) and that has been proposed to be the default activity pattern of the cortical network (*2*). This oscillatory rhythm generates extracellular currents and electric fields (EFs) that are prominent enough to be extracellularly measured in the conductive medium [local field potentials (LFPs)] and also from the skull surface [electroencephalograms (EEG)]. These EFs generated by neuronal activity, in turn, induce changes in the activity of neurons (*3*, *4*). In other words, the electric environment generated by neuronal activity has a feedback effect on neuronal activity that shapes and modulates the final network activity (*3*–*8*).

This so-called ephaptic coupling is a well-known phenomenon first demonstrated in the 20th century in studies showing that the electrical activity of one nerve may influence the firing of a second adjacent nerve (*9*). Similarly, cells that are not synaptically connected can interact by means of EFs through the conductive medium (*4*). Thus, ephaptic coupling between neurons can end up synchronizing networks with a detectable feedback effect on oscillatory patterns, especially evident in the case of hippocampal epileptic discharges (*10*).

During slow oscillations, almost the entire neuronal population is involved in a largely synchronized pattern that generates EFs with periodic waveforms (*3*, *8*). Given the impact of slow frequencies on ephaptic interactions (*4*), there is a possibility that slow rhythms may evoke particularly powerful EF effects. Exogenous EFs can induce changes in the firing timing of neuronal populations, thus implying that field effects can modulate oscillatory activity (*11*). An exogenous EF can entrain subthreshold activity and spike trains if oscillating at a slow rhythm (1 Hz) (*4*). These results suggest that EF effects, even if small, can be amplified by network dynamics (*12*). Previous work in cortical ferret slices demonstrated that exogenous EFs mimicking endogenous EFs are able to entrain oscillatory network activity (*3*), supporting the idea that endogenous EFs are not a mere idling of neural activity (*6*, *7*). Most studies that addressed the effect of EFs on neuronal activity by applying exogenous EFs (*3*, *7*, *11*–*14*) report a critical effect of weak EF stimulation on spiking due to its impact on membrane voltage. In particular, in vitro experiments on hippocampal slices demonstrated that the application of weak EFs influenced oscillatory activity (*11*). Slow oscillations represent a suitable testbed to study synchronization across cortical columns mediated by ephaptic interactions. As an emergent property of the network, slow oscillations are more sensitive to field effects than single neuron activity (*8*), because a small change in membrane potential (i.e., 0.5 mV) in individual neurons can result in a noticeable change in emergent slow oscillatory frequency (*3*, *12*) through recurrent amplification by the network. The existence of a feedback loop between neuronal activity generation of EFs and impact of those EFs on the neuronal activity implies a difficulty disentangling these elements. Here, we explore this question experimentally and in a computer model.

To investigate the effect of endogenous EFs on network activity and on intercolumnar interactions, we used a cerebral cortex slice preparation that allowed us to manipulate a number of parameters that cannot be isolated in vivo. We first show EF propagation in the cortical network, the generated EF gradient, and we test several experimental manipulations that can disturb it. We then provide evidence that such EF impact on oscillatory frequency between adjacent columns displays properties of electric dipole interaction. Last, we present a computational model showing that populations of electric dipoles can account for these results and make predictions for how endogenous EFs may affect slow oscillations in the intact brain.

## RESULTS

### Endogenous fields in synaptically disconnected networks

**Fig. 1:**
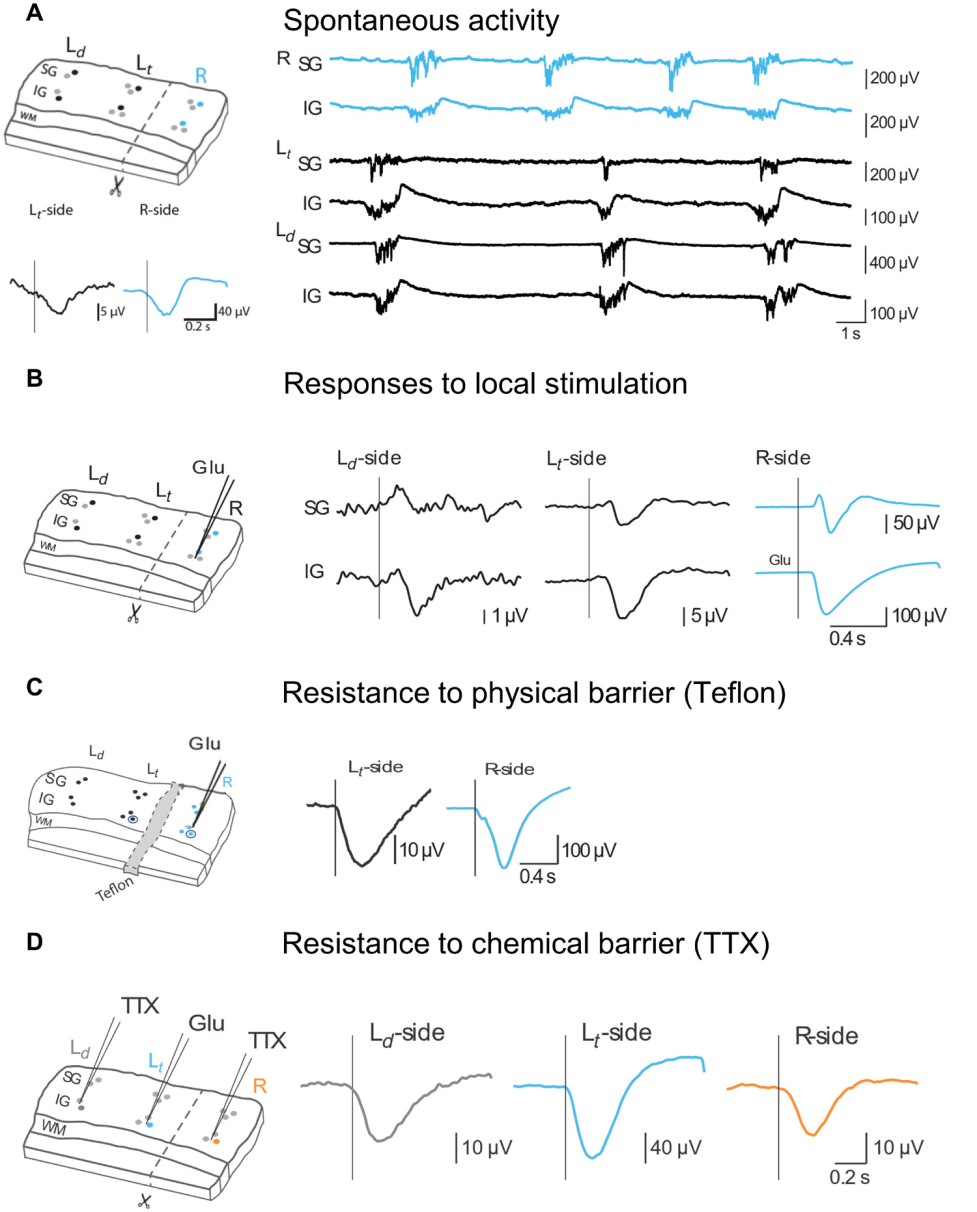
Endogenous-field propagation of cortical slow waves. (**A**) Left: Sectioned cortical slice scheme (top) and Up state (*n* = 15 waveform average) (bottom). Right: Slow oscillations recorded from supragranular (SG) and infragranular (IG) layers at the six different electrodes indicated on the scheme with dark-color circles. Right side of the slice in blue (R), left side of the slice in black. (**B**) Left: Sectioned cortical slice scheme. Right: Glutamate-induced responses on R-side (blue) and EF waves at L-side (black) recorded in a particular slice (*n* = 30 waveform averages). Vertical black lines represent onset time obtained from the response detection (see Materials and Methods). (**C**) Recordings in the presence of a thin Teflon barrier (scheme) in between the two hemislices, in response to a glutamate-induced responses at R-side (blue) (*n* = 33 waveform average from one slice). (**D**) Resistance to application of TTX to both sides (scheme). The response is shown following a puff of glutamate on the left side (*n* = 20 waveform average from one slice). WM, white matter; L*_d_*, diodes on the left; L*_t_*, triodes on the left; R, right; Glu, glutamate. Recording traces and waveform average were recorded at electrodes represented with dark-color circles on the schemes; light-color circles represent electrodes from which no trace or waveform average is displayed.

Slow oscillations similar to those observed during slow-wave sleep (*1*) were recorded from 69 visual cortical slices (*15*). Spontaneously generated slow oscillations had an average frequency of 0.25 ± 0.02 Hz. Slow oscillations propagate along the cortical slice (*15*–*17*) via local synaptic connectivity, reproducing features of in vivo propagation (*18*). To investigate to what extent the activity could have a nonsynaptic—but ephaptic—propagation, we performed a complete cut of the slice perpendicular to the cortical layers resulting into two different networks synaptically disconnected that could be simultaneously recorded (Fig. 1A). In this manner, both left and right hemislices remained in contact, firmly adhered at the bottom of the interface chamber formed by filter paper, while we recorded the network activity with a 16-channel array from the surface (see Materials and Methods; fig. S1). The array also allowed us to carry out measures at fixed distances during the various experimental manipulations. Sectioning the slice resulted in two independent networks that acted as two independent oscillators. Hence, different oscillatory patterns could emerge at each side of the cut (Fig. 1A and fig. S2B). We observed the propagation of the EFs generated by the spontaneous Up states across the cut, albeit with a decay in the amplitude (Fig. 1A, inset; see below).

To better determine the field’s temporal and spatial propagation, we next triggered network responses to have a temporal control of their occurrence. We did this chemically (with local application of 10 to 20 pl of 0.5 mM glutamate; *n* = 10 slices) at a frequency similar to the spontaneous slow oscillations (~0.25 Hz) (Fig. 1B). As observed for spontaneous events, induced events also electrically propagated across the cut with a decay in amplitude. LFP recordings from both sides of the cut showed that responses originating at the R-side of the slice could be recorded across the cut (at the L-side) with a decrease in amplitude that increased with distance (fig. S2, C to F). The response in the L-side had an amplitude that corresponded to the 4.3 ± 0.01% of the original response at the R-side (separated by the cut). The peak amplitude decreased with distance from the original site: Field’s amplitudes were 6.70 ± 1.06 and 3.54 ± 0.82 μV at the L*_t_*-side and at the L*_d_*-side (1.5 and 3 mm apart from the origin site, respectively), while the amplitude of the original evoked glutamate response was 529.80 ± 289.16 μV at the R-side [means ± SEM from infragranular (IG) layers] (fig. S2F; population average values from 10 slices). The decay in the amplitude of the events with distance illustrated in Fig. 1 (B to D) demonstrates the gradients in extracellular voltage that should cause electric dipoles in neurons (see below, section “Relevance of cortical dipoles to EF entrainment of oscillations”).

### Endogenous fields do not result from chemical propagation nor neuronal firing

To rule out the possibility of glutamate diffusion across the cut, we explored the nonsynaptic propagation of glutamate-induced responses across a piece of polytetrafluoroethylene (PTFE; i.e., Teflon) between both sides in five slices (Fig. 1C). We observed that glutamate-induced responses still propagated, although they were reduced to 9 ± 0.01% at the L*_t_*-side (1.5 mm) under this condition. This reduction was not significantly different to the one observed when both sides of the slice were attached (4.3 ± 0.01%, Sign test, *P* = 0.06), strongly suggesting that there is no glutamate diffusion across the cut of between both sides. The insulating properties of Teflon also ruled out the possibility of propagation through endogenously released K^+^ across the cut.

To explore whether the cut per se had any effect on the EF propagation, we then blocked synaptic activity by means of tetrodotoxin (TTX) application on the same piece of cortex where the glutamate responses were evoked. With this, we compared the EF propagation at equidistant (1.5 mm) points in the array: on the pharmacologically isolated site with 30 μM TTX (L*_d_*-side) and on the physically (R-side) synaptically disconnected networks (Fig. 1D). Postsynaptic glutamate responses were induced at the L*_t_*-side (blue traces), while local TTX applications were delivered at the L*_d_*-side (gray traces). The result was that a response with an average amplitude of 11.55 ± 2.17 μV propagated to the L*_d_*-side and similarly shaped responses with an average amplitude of 18.63 ± 2.26 μV propagated to the R-side (Sign test, *P* = 0.18; *n* = 9 slices). These similarities were maintained when blocking synaptic activity in the physically disconnected network, again by applying locally TTX (R-side) (11.83 ± 1.1 μV at the R-side and 9.38 ± 1.82 μV at the L*_d_*-side; Sign test, *P* = 0.45; *n* = 7 slices) (Fig. 1D, orange and gray traces). Overall, these results show that EFs can be generated by spontaneous events or evoked responses, without requiring amplification by local synaptic activity, as shown in Fig. 1A. The similarity between the waves recorded from the pharmacologically disconnected network (L*_d_*-side) and from the physically disconnected (R-side) network suggests that the cut hardly had any effect on the detected EF activity.

### Endogenous fields affect synaptic activity and rhythmicity

**Fig. 2:**
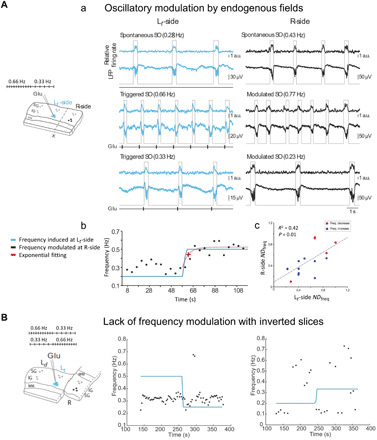
Frequency modulation between two synaptically disconnected networks. Oscillatory frequency modulation by EFs originated on the synaptically disconnected column (**A**) (a) Relative firing rate, LFP recording and Up and Down state detection (gray lines) obtained at both sides of the cut (see Materials and Methods). Spontaneous activity (top) and two “triggered” oscillatory frequencies: 0.66 and 0.33 Hz (middle and bottom, respectively) at L*_t_*-side; the modulated slow oscillations on the R-side changed to 0.77 and 0.23 Hz, respectively. Traces from both sides are aligned on time and plotted in consecutive order (from top to bottom) as they were recorded. SO, slow oscillation; a.u., arbitrary units. (b) Exponential fitting for a particular change in frequency [top of (A)] displaying the glutamate application frequency (blue line); the modulated slow oscillation frequency at R-side (black dots) and its exponential fitting displaying the τ (red cross). (c) Dispersion plot of the *ND*_freq_ on both sides of the slice. Increases in frequencies, blue; decreases in frequencies, red (*n* = 15 variations, 10 slices). (**B**) From left to right: Schematics of the stimulation with glutamate on the left section of the slide and investigation of the modulation on the right side with an inverted slice. Above, the schematics of stimulation frequency, decreasing (0.5 to 0.25 Hz; middle) or increasing (0.2 to 0.3 Hz; right side). In blue, the stimulation frequency with glutamate application. Notice the absence of modulation on oscillatory frequency in two different slices.

It has been suggested that EFs have an effect on the neuronal networks that generate them, and as a result, there is a feedback loop between EFs and synaptic activity (*6*). Given the difficulty of teasing apart synaptic activity from EFs, to study this feedback interaction, most studies have used exogenous EFs stimulation through two parallel electrodes that create an EF (*3*, *7*, *11*, *12*, *14*, *19*) or electrical stimulation inside and outside individual cells (*4*) to demonstrate that external fields are able to entrain neocortical network activity. However, isolating synaptic activity from EFs still remains an experimental challenge. Here, we investigated whether spontaneous slow oscillations in one network could be modulated or entrained by the EFs generated by slow oscillations in the adjacent network. Slow oscillations are considered the largest spatially synchronized rhythmic pattern in the brain. External DC stimulation mimicking EFs from slow-wave activity recorded in vivo can entrain spontaneous activity in cortical slices eliciting the same slow oscillation pattern (*3*), suggesting that EFs could guide the orchestration of oscillatory activity. To further explore the impact of endogenous EFs on spontaneous rhythmic activity, we used glutamate-evoked Up states to elicit control over the frequency of the Up states (*n* = 10 slices). Thus, slow oscillations at different frequencies were evoked on the L*_t_*-side, called “triggered” slow oscillations, which are represented by the blue Up/Down detection traces on Fig. 2A (a). By doing this, we explored the impact of these “triggered” slow oscillations on the spontaneous slow oscillation frequency of the R-side, called modulated slow oscillations (black Up/Down detection traces). This manipulation resulted in a frequency variation across the cut. Increasing (decreasing) the frequency of the “triggered” slow oscillations on the L*_t_*-side induced a parallel increase (decrease) in the modulated slow oscillation frequency on the R-side (Fig. 2A, a, and fig. S3). With this, we demonstrated that the L*_t_*-side network is able to entrain the R-side one, suggesting that two synaptically disconnected networks can be loosely synchronized by EFs. It should be noted that the slow oscillations did not reach exactly the same frequency on both sides of the cut: R-side traces (with a frequency of 0.43, 0.77, and 0.23 Hz—from top to bottom) are aligned on time with their respective L*_t_*-side (with a frequency of 0.28, 0.66, and 0.33 Hz; Fig. 2A, a).

This loose synchronization was also manifested in the fact that the modulated change in frequency took some seconds to occur (Fig. 2A, b), specifically an average of 17.24 ± 4.86 s for increasing frequencies, *n* = 10 frequency variations; 17.53 ± 10.13 s for decreasing frequencies, *n* = 5 frequency variations; from a total of 10 slices (see Materials and Methods). We speculate that this was the time taken by the emergent activity in the modulated network to get organized in a situation of enhanced excitability. Because the “triggered” and the modulated slow oscillation cycles (at L*_t_*-side and R-side, respectively) did not exactly reach the same frequency, normalized differences of frequency variations (*ND*_freq_) (see Materials and Methods) were quantified to compare the frequency changes observed in both independent networks when varying the Up state induction periodicity at the L*_t_*-side. The dispersion plot in Fig. 2A (c) represents the *ND*_freq_ for the triggered slow oscillation frequency on the L*_t_*-side versus the *ND*_freq_ of the entrained slow oscillations on the R-side. The result was a linear relationship between both *ND*_freq_ with linear regression values (*R*^2^ = 0.42, *P* = 0.01 for 15 frequency variations; in a total of 10 slices). Of 16 slices in which this protocol was tested, the modulation was detectable in 10 of them (Fig. 2A, c). These results demonstrated that the frequency variation on the R-side was the consequence of the frequency variation on the L*_t_*-side and that there is a trend toward converging in oscillatory frequency, a feature that we will replicate and quantify in our computer model (see below). Then, we can conclude that EFs generated from slow oscillations are able to modulate and eventually entrain a synaptically disconnected network and thus might have a role in the synchronization of neighboring cortical columns.

### Relevance of cortical dipoles to EF entrainment of oscillations

To determine whether the EF propagation might be due to dipoles, we have tested the robustness of the interaction to the laminar orientation. Previous studies have described the influence of the cortical laminar structure in the generation and propagation of slow oscillations (*15*–*17*, *20*). However, how the laminar orientation of the cortex affects the EF propagation remains an open question. To explore the influence of the laminar orientation in the spread of EF and the entrainment effect described above, we gently overturned the network on the R-side of the cut by 180° so that supragranular (SG) layers were next to IG layers and vice versa (*n* = 7 slices). In this manner, the two separate cortical networks were still tightly in contact, but with opposite laminar orientation (Fig. 2B and fig. S4). Under this condition, the recorded EF waves were not influenced by the distribution of the cortical layers, and we observed a similar decay in amplitude over distance to 23.13 ± 9.04 and 18.72 ± 3.91% when spontaneous Up states and induced glutamate responses (respectively) originated on the R-side propagated across the cut to the L*_t_*-side (1.5 mm) (fig. S4A). However, these EFs were not able to modulate the slow oscillations at the adjacent inverted network. Triggering the slow oscillation at different frequencies in one side of the cut (at either SG or IG layers, *n* = 5 frequency variations; in a total of three slices) did not entrain slow oscillations on the other side (Fig. 2B and fig. S4B). These results show that two adjacent synaptically disconnected networks can only be synchronized by EF when their laminar pattern is similarly oriented, consistent with the fact that the EF interactions might occur through electric dipoles, which must be parallel.

**Fig. 3:**
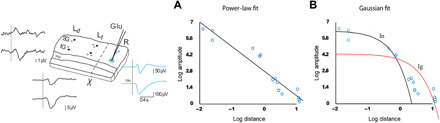
Decay of the amplitude of the EF response with distance, compatible with electric dipoles. Left: Schematics illustrating the gradient in the amplitude of the responses. (**A**) Log-log representation the EF waves amplitude at the 10 different locations on the L*_t_*-side following glutamate injection at the R-side. The straight line indicates the best linear regression fit in this representation, which corresponds to a power-law decay with distance (1/*r^a^*, with *a* = 2.1) (m.s.e. (mean squared error) = 0.41). (**B**) Same representation with two Gaussian fits, which correspond to the solution of the diffusion equation. The two fits were calculated according to the linear error (black, m.s.e. = 105.12) or the error calculated in log-log scale (red, m.s.e. = 1.74).

To further test the presence of electric dipoles, we have followed previous studies (*21*, *22*) and measured the fall-off of the evoked potential as a function of distance, which should follow a 1/*r*^2^ profile, according to Coulomb’s law [see (*23*)]. This was tested by measuring the “echo” of glutamate-induced responses (Fig. 1B) as a function of distance (Fig. 3). The estimation of the decay with distance matched the 1/*r*^2^ predicted by electric dipoles (Fig. 3A). We also tested the decay predicted by ionic diffusion, which should theoretically have a Gaussian profile, but such a profile could not lead to acceptable fits to the data (Fig. 3B). We therefore conclude that the decay of the EF response with distance is consistent with the power-law profile predicted by electric dipoles and that ionic diffusion again cannot explain our recordings.

### Modeling intercolumnar synchronization by EFs

To obtain a more quantitative understanding of the mechanisms involved in the phenomena described so far, we developed a mean-field model of a column consisting of two synaptically connected populations of excitatory and inhibitory neurons. The two columns are coupled solely through the EF generated by their activities, assuming electric dipole interactions (see Materials and Methods). The amplitude and sign of the electric interaction was estimated from a cable model adjusted to the potential gradient measured in the slice, leading to an estimate of the EF-induced membrane depolarization (fig. S5). A leaky-integrate-and-fire (LIF) model was used to estimate the effect of “dendrite polarization” on the firing rate of a single neuron. We assumed that only pyramidal neurons were sensitive to the voltage gradient through their apical dendrite. Notice that even if the depolarization is very small (fig. S5B), it is amplified by recurrent interactions, and this is precisely what the mean-field can account for.

**Fig. 4:**
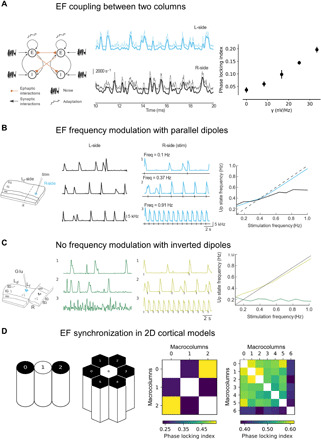
Model of the entrainment of slow oscillations from EF interactions between dipoles. (**A**) Left: Scheme of two excitatory populations mutually coupled solely through the EF (no synaptic connectivity). Middle: The occurrences of the Up states synchronize weakly because of the EF interaction. Right: Dependence of the PLI on the coupling strength γ (membrane depolarization induced on the “receiving” population per one spike per second of the activity of the “source population”). As a measure of variation, we calculated the SD across 10 repetitions of the simulation for each value of gamma. (**B**) Left: Scheme of the stimulation protocol simulated by the model. In the model, glutamate injection (Stim) is applied on the R-side (blue) triggering periodic Up states at the R-side and producing EF that affects activity at the L-side. Two middle panels: Sample traces of excitatory population rate at both sides of the slice at three different stimulation frequencies. Right: Frequency of the Up states at both sides of the slice as a function of stimulation frequency. L-side entrains to the R-side by means of the EF (ephaptic) coupling. (**C**) Same arrangement as (B), but with inverted dipoles, simulating the inverted slice experiments. (**D**) Left schemes: Topology of the network in one dimension and two dimensions. Color plots: PLI for the different populations. The populations in black received synchronous external stimulation, and the neighboring populations interacted through EF. The PLI is indicated by color in each plot (see scale). 2D, two-dimensional.

To reproduce the experimental protocol (Fig. 2 and fig. S2), we modeled the L- and R-side of the slice by identical excitatory/inhibitory mean-field models generating spontaneous Up/Down oscillations. We periodically triggered Up states in the R-side by current injections (square wave) to the excitatory population. This led to the appearance of regular Up states at the R-side site and associated periodic EF. This EF entrained the frequency of Up states generated in the other network. The two populations weakly synchronized; that is, the Up states of the two populations coincided more often than by chance (Fig. 4A). We quantified the level of such synchronization using the so-called phase locking index (PLI). We found that with realistic parameters of the model, we obtained small but significant PLIs, the values of which increase with increasing coupling strength (Fig. 4A, right) supporting the idea that EF coupling can contribute to the synchronization of neighboring columns. As a measure of significance of the PLI differences between different oscillator toplogies, we calculated the mean and the SEM of PLI values for all possible pairs of macrocolumns.

The frequency of the Up/Down oscillations at the R-side modulated Up/Down oscillations across the cut (L-side, Fig. 4B), but without reaching identical frequencies or perfect synchronization, which we call here “loose synchronization.” This finding is consistent with the results of an analogous protocol performed in the slices, providing further evidence that the coupling between the two sides of sectioned slice is mediated by the EFs (Fig. 2).

To further test the role of dipolar interactions, we have simulated the inverted slice experiment by inverting the EF coupling between the dipoles, which were then oriented according to opposite directions (Fig. 4C). The coupling was hyperpolarizing in this case (fig. S5B, red line), because the gradient was inverted compared to the normally oriented slice (compare with Fig. 1B). Similar to the experiments, this configuration yielded no frequency modulation in the neighboring network (Fig. 4C), consistent with the inverted slice experiments. Figure S4 shows reduction in amplitude of EF waves (fig. S4A) and lack of synchronization/coupling between both sides (fig. S4B). Because the coupling is inverted, we can speculate that the amplification through recurrent interactions does not occur and the coupling remains negligible.

Last, because the macrocolumns in the visual cortex in vivo are organized in a two-dimensional sheet, we used the model to predict the strength of the synchronization in a one-dimensional chain, modeling in vitro slice experiments and in a two-dimensional hexagonal configuration of macrocolumns (both schemes are shown in Fig. 4D), consistent with cortical in vivo anatomy. In both models, the neighboring macrocolumns interact by means of the EF (ephaptically) only. Synchronous Up/Down transitions were evoked by the external stimulation to selected macrocolumns of the network: Only macrocolumns located at the edge of the network (black circles/hexagons in Fig. 4D) received the common stimulus. The central macrocolumns were entrained by the pairwise interactions with their neighbors, and they did not receive external inputs. We calculated the PLIs between the macrocolumns in both topologies (Fig. 4D, color graphs). The macrocolumns at the edges of the networks showed high PLIs between each other as a result of external stimulation [PLI: chain, 0.54 (*n* = 1 pair); hexagon, 0.567 ± 0.007 (means ± SEM, *n* = 15 pairs)]. However, the central macrocolumns were also partially synchronized to the simulated macrocolumns (and indirectly to the external stimulation) by the effect of the EF, but the PLIs with the rest of the network were lower [PLI: chain, 0.238 ± 0.004 (means ± SEM, *n* = 2); hexagon, 0.383 ± 0.006 (*n* = 6); see Fig. 4D, color graphs]. We found considerable difference between the mean PLIs of the central macrocolumn and the rest of the network in the one- and two-dimensional topologies. This difference is expected because of the higher number of (synchronous) neighbors in the hexagonal topology (two-dimensional, six neighbors) and chain topology (one-dimensional, two neighbors; see Fig. 4D). The model thus predicts that ephaptic interactions should be stronger in a two-dimensional sheet of cortical columns.

## DISCUSSION

In a preparation of cortical brain slices spontaneously eliciting slow oscillations (*2*, *15*, *24*), we found that slow waves generate EFs that propagate independently of synaptic transmission within the cortical tissue (Fig. 1), suggesting that these endogenous fields can participate in the physiological synchronization of cortical columns. In this study, we have demonstrated that the oscillatory activity of one cortical network modulates that of the neighboring cortex, even when synaptically disconnected (Fig. 2). This is, to our knowledge, the first evidence that endogenous nonsynaptic mechanisms contribute to the coupling of neuronal populations across cortical columns, thus having an impact on information processing and synaptic plasticity. EFs in the brain emerge from the contribution of extracellular currents, the spatial alignment of neurons, and their synchronized activity being the major determinants of the EF measured in neural tissue (*6*, *25*, *26*). In the work presented here, we included physiological network synchronized patterns such as slow oscillations, epileptiform discharges, or chemically evoked responses to study the impact of EFs. Although further experimental evidence is needed to fully understand the contribution of the different sources of the EFs (*26*), our experimental approach demonstrates that the EFs generated by the synchronized population activity can be effectively dissected from the synaptic interactions that generate them (Fig. 1). Cutting the slice into two pieces perpendicularly to the cortical layers yielded two independent networks with independent oscillatory patterns. The population activity originating on one side of the cut can be recorded as electric potentials caused by the EF propagation, on the other side (Fig. 1A). Previous work using similar approaches reported that the nonsynaptic propagation of epileptiform activity might be caused by the endogenously released K^+^ (*27*); however, in our experiments with a physical barrier or with a gap between the two pieces of the slice, we demonstrated that there was no diffusion of K^+^ (nor of glutamate) across the cut. Moreover, the resulting propagation speed across the cut of the epileptiform responses was three to four orders of magnitude faster (388.88 ± 171.5 mm/s) than the diffusion speed of K+ (0.05 to 8 mm/s) (*28*), which is consistent with the nonsynaptic propagation of neural activity reported by recent in vitro studies (*29*, *30*). Furthermore, our results showed that the decay of the amplitude of EFs with distance did not follow a Gaussian as chemical diffusion does (Fig. 3), but a power law, describing the decay of EFs and that predicted for an electric dipole. We can therefore conclude that diffusion is not the mechanism responsible for the nonsynaptic propagation observed in our experiments.

We found that EFs decayed with distance when traveling across the cut between both disconnected networks (Fig. 1B and fig. S2). In the first instance, this is explained by the fact that extracellular voltage magnitudes are reduced with the inverse of the distance from the origin source to the recording point (*26*). This amplitude decay and the delays discard the possibility that the propagated waves were artifacts due to cross-talk effects between the different electrodes. Given that slow frequencies attenuate less and showed larger spatial correlation than fast frequencies within the neural tissue (*19*, *31*), studying of slow oscillations seems to be a good approach to further explore the conductive properties of neural tissue. The electrical activity of a population of neurons leads to changes in extracellular ion concentration that creates a global influence in active networks. Such influence represents the contribution of EFs to neuronal activity and has been posited to modulate the excitability of neurons contributing to neuronal synchronization (*13*, *32*–*35*). External EF stimulation has been the strategy most often used to investigate the feedback interactions between endogenous EFs and synaptic activity (*3*, *7*, *11*, *14*, *36*). In the work presented here, the isolation of EF and synaptic activity allowed a direct exploration of their interactions. In particular, we showed, using a computational model, that the potential gradients measured experimentally are sufficient to explain the ephaptic effects, assuming electric dipole interactions.

The hypothesis that cerebral cortex forms electric dipoles is commonly used in EEG source localization methods (*37*) and is supported by experiments where the LFP was recorded together with an intracellular recording (*21*), where a fall-off as 1/*r*^2^ was reported. This was also confirmed by detailed computational models of pyramidal neurons (*38*). Furthermore, a reanalysis of published data from various sources showed that the fall-off of 1/*r*^2^ generally applies to distances larger than 50 μm from the neuronal sources (*22*). The present results are completely compatible with these estimates, as the dipolar effects that we reported here were at distances of the order of 100 μm and more.

Thus, our experimental findings and model demonstrate that EFs might be able to synchronize neural activity by modulating its timing, given that a slight depolarization in individual neurons facilitates, at the population level, the earlier initiation of subsequent Up states. The time delay observed between both networks when we changed the frequency of the Up/Down cycle (Fig. 2A, b) would reflect the time that the network needs to adapt to the new frequency facilitated by the EF activity coming from the adjacent network. Also, we observed that to reach such frequency modulation, both networks need to have their cortical layers arranged in their correct order, because rotating one side of the sectioned slice abolished the frequency modulation (Fig. 2B). This is entirely compatible with interacting dipoles, which must be oriented in parallel to interact electrically. We reproduced these experimental findings using a mean-field model of the Up/Down oscillations generated by neural populations interacting through electric dipoles. In this model, the electric dipole associated with an Up state caused a depolarization in the membrane potential of the excitatory population on the L-side. This depolarization, in turn, increased the probability of generating an Up state, thus influencing Up/Down transitions. Importantly and similarly to the recorded slices, the resulting entrainment was not perfect; that is, there is no exact temporal relationship between the Up states in L- and R-sides nor are the frequencies of their occurrences exactly matched. Because the magnitude of EFs and its decay with distance are matched to experimental data, these modeling findings strengthen the evidence that the EF can functionally couple synaptically dissociated networks through electric dipole effects. To corroborate this conclusion, we also implemented two networks that oscillate spontaneously between Up and Down states and showed that they can mutually adjust their activities through the EF, leading to an increase in synchrony. The entrainment was progressive because the EF interaction only takes place during the Up states, although we did not attempt to quantitatively model the observed experimental delay.

The consistency across experiments and modeling constitutes strong evidence that neighboring cortical columns can synchronize through ephaptic coupling between electric dipoles. More generally, we expect that the effect found here should be even stronger in two-dimensional networks of cerebral cortex in vivo where pyramidal neuron dipoles are arranged in parallel and can thus receive EF-mediated depolarization from their broad neighborhood. In addition, the interaction can be further enhanced by the positive feedback between synchrony, inducing stronger EFs, which further increases synchrony, and so on. We demonstrated this in a two-dimensional network model with population dipoles in hexagonal connectivity, which produced considerably higher synchronization measures (PLI) compared with the one-dimensional topology consistent with in vitro experiments. Therefore, we suggest that the effect should be stronger in vivo compared to slices.

Although ephaptic coupling has been known since the 1940s, its role in physiological activity has been considered near to negligible. In our computational model, we see that for an asynchronous firing neuron (similar to an awake state), only 1% of firing rate would be affected by ephaptic coupling (not shown). Only for strongly synchronized firings, such as epilepsy, has EF coupling been known to have a role in the network synchronization (*5*, *10*, *27*, *32*, *39*). However, here, we show that during normal, physiological synchronized activity, for instance, slow wave sleep, the synchronization of the population is such that there should be a notable impact of ephaptic coupling in the synchronization of neighboring cortical areas. Moreover, because ephaptic coupling is nearly instantaneous, it may dominate over the synchronization mediated by synaptic connections for high-frequency signals. Ephaptic coupling was used, for example, to explain the fast propagation of epileptiform activity through hippocampal networks (*40*). Similarly, in vivo experiments performed in barrel cortex of rodents identified fast oscillations (>200 Hz) in LFPs that were coherent across multiple barrels with submillisecond precision (*41*). Because the synchronous oscillations were established nearly instantaneously, it has been suggested that the synchronization was mediated by gap junctions or EF interactions (*42*, *43*). Our experiments and models of the visual cortex also support EF-based mechanisms, which are here mediated by electric dipole interactions between adjacent columns. Future studies should investigate its possible impact on information flow in cerebral cortex in vivo.

## MATERIALS AND METHODS

### Experimental design

The objectives of the study were (i) to determine whether endogenously generated EFs could be recorded; (ii) if recorded, whether they modulated spontaneously generated oscillations; (iii) to investigate the mechanism of action of the modulation; and (iv) to model the experimental results and further investigate mechanisms and impact on the network. To do this, 69 ferret cortical slices were used in different series of experiments including a diversity of experimental maneuvers.

### Preparation and maintenance of slices

Ferrets (4 to 10 months old, either sex) were anesthetized with sodium pentobarbital (40 mg/kg) and decapitated. The entire forebrain was rapidly removed to oxygenated cold (4° to 10°C) bathing medium. Ferrets were treated in accordance with the European Union guidelines on protection of vertebrates used for experimentation (Directive 2010/63/EU of the European Parliament and of the council of 22 September 2010). All experiments were approved by the local ethics committee.

Coronal slices (0.4 mm thick) from visual cortex (areas 17, 18, and 19) were used. A modification of the sucrose substitution technique was used to increase tissue viability. During slice preparation, the tissue was placed in a solution in which NaCl was replaced with sucrose while maintaining osmolarity. After preparation, the slices were placed in an interface style recording chamber (Fine Sciences Tools, Foster City, CA) and superfused with an equal mixture in volume of the normal bathing medium, artificial cerebrospinal fluid (ACSF), and the sucrose-substituted solution, for 15 min. Following this, ACSF was switched into the recording chamber, and the slices were superfused for 80 min; ACSF contained 126 mM NaCl, 2.5 mM KCl, 2 mM MgSO_4_, 1 mM Na_2_HPO_4_, 2 mM CaCl_2_, 26 mM NaHCO_3_, and 10 mM dextrose and was aerated with 95% O_2_ and 5% CO_2_ to a final pH of 7.4. Then, a modified ACSF was used throughout the rest of the experiment; this was similar to the normal bathing medium except for different levels of the following: 4 mM KCl, 1 mM MgSO_4_, and 1 mM CaCl_2_. Bath temperature was maintained at 34° to 36°C.

To separate the synaptic from the EF (nonsynaptic) activity, a complete cut of the slice perpendicular to cortical layers was performed with a scalpel blade. The cut was done while the slices were in the interface chamber, allowing the two sides (left side, L-side; right side, R-side) to remain either tightly in contact without discontinuity between them or with a gap (~300 μm) between both sides. In nine slices, a piece of PTFE (i.e., Teflon) ~400 μm thick and ~200 to 400 μm long was positioned between both sides. The slices remained mechanically stable and firmly adhered at the bottom of the chamber formed by filter paper. At the end of every experiment, we removed the two sections of each slice from the filter paper, confirming that they were indeed completely separated, which occurred in all cases.

### Electrophysiological recordings

Extracellular LFP recordings were obtained with 16 gold electrodes plated with platinum black disposed on a recording grid (fig. S1). The grid including an array of holes was designed and fabricated using SU-8 negative photoresist or polyamide as described by Illa *et al.* (*44*). Electrode impedances and phases were tested with known signals before the recordings for each array, excluding the possibility of delays or distortion that differences in electrode coating could induce.

The recording array was placed on top of the slices, and 16 simultaneous recordings were obtained. The electrodes were grouped in six recording spots: There were two to three electrodes (separated by 200 mm) (diodes or tritrodes, respectively), half of them recorded from SG and the other half recorded from IG layers, as well as from three different cortical columns [diodes/tritrodes were 750 mm apart in the vertical axis and 1.5 mm apart in the horizontal axis (fig. S1)]. In the sectioned slices, 10 electrodes recorded at the L-side of the cut (L*_d_*-side refers to the electrodes on the left conforming the diodes, and L*_t_*-side refers to the electrode on the left conforming the tritrodes) and 6 electrodes at the R-side. To simplify, one representative electrode was selected from each diode/triode to study synaptic and EF activity.

Neural activity was referenced to an Ag/AgCl electrode placed at the bottom of the chamber in contact with the ACSF. Unfiltered signals were acquired with a Multichannel System amplifier and digitized at 10 kHz with a Power1401 interface and Spike2 software (CED, Cambridge, UK). No filters were added during the recording stage to avoid signal distortion.

### Pharmacological manipulations

Glutamic acid (glutamate, 0.5 mM) from Sigma-Aldrich and TTX (30 μM) from Tocris were applied by delivering a brief pulse of nitrogen to a glass micropipette containing the drug (10 to 20 pl) (*15*). Bicuculline methiodide (2.4 to 3 μM) from Sigma-Aldrich was bath-applied to transform the spontaneous slow oscillations in epileptiform activity (*45*), generating large responses strongly evident across the cut. It should be noted that in the interface chamber used, it takes around 20 min to get a stable concentration in the bath, so all measurements were taken after this period.

### Data analysis

All analyses were performed offline with Spike2 software (CED, Cambridge, UK), plus custom-written or MATLAB toolbox scripts (The MathWorks Inc. Natick, MA). All average values are presented as means ± SEM. Kolmogorov-Smirnov test was used to test for normality; as none of the samples followed a normal distribution, a nonparametric (Sign test) were used to determine significance.

#### Up state detection analysis

Up and Down states were detected as previously described (*45*, *46*). Relative firing rate or was used as a measure of the population firing rate based on the multiunit activity (MUA) spectrum. High-frequency components of the extracellular recording can be seen as a linear transform of the instantaneous firing rate of the neurons surrounding the electrode tip. Theoretical studies show that the normalized MUA spectrum provides a good estimate of the population firing rate, given that normalized Fourier components at high frequencies have densities proportional to the spiking activity of the involved neurons (*47*). For that reason, the spectrum of the power between 200 and 1500 Hz is considered to be a good estimate of the firing of the neuron population (*47*). This estimation has been previously used (e.g., *45*, *46*). For the identification of Up and Down states, three different time series were obtained from the signal: the slow oscillation envelope from the slow LFP deflection, the MUA from the population firing rate (*47*), and the envelope of gamma rhythm variance (*48*). A linear combination of the three-time series was obtained in which the contribution of each time series was calculated by principal component analysis. Up states were detected by setting a threshold in this processed time series.

#### EF wave detection and waveform-average analysis

Signals were down-sampled at 500 Hz and low-pass–filtered at 100 Hz. To detect the EF waves in the synaptic disconnected network, average waveforms of the LFP signal across repetitions of Up states in one side of the cut were obtained at each recording point. Cases where the studied EF response at the adjacent side of the slice overlapped on time with spontaneous Up states (Figs. 1A, right-most Up states) were discarded from the averages.

The Up state onset time obtained from the Up/Down detection was considered as reference time for all detected waves, and average waveforms were calculated from a time window of 1.1 s, between 0.3 s before and 0.8 s after this onset time. Each 1.1 s LFP segment was adjusted at zero voltage offset by subtracting the mean voltage of the 0.3 s before the onset time. Amplitude was considered the voltage difference between this offset and the minimum peak from the final waveform average.

For the speed analysis, delays were expressed as a matrix of relative time lags between detected onsets of EF waves by setting a common threshold in the waveform-average LFP for all of the channels. The speed was estimated by dividing these delays by the electrode distances.

#### Modulation kinetic analysis

From the Up/Down detection method previously described, Up/Down cycle time series were obtained and values at 3 SD from its mean were considered outliers. These time series were subsampled by defining bins of fixed width, in a way that each bin entailed at least one Up/Down cycle. Samples of each bin were averaged resulting in a constant sample frequency across each time series. Last, an exponential fitting was adjusted on the modulated slow oscillations (at the R-side, where no glutamate was applied) from the time when the glutamate application (at the L-side) changed the frequency (Fig. 2A,b).

The exponential fitting was adjusted according to

$$y=a(1-e^{-\frac{1}{b}t})$$

for increasing frequencies, and to

$$y=a(e^{-\frac{1}{b}t})$$

for decreasing frequencies.

As mentioned above, the start point of the exponential was set at the time where the glutamate application (at the L-side) changed the frequency. Thus, *b* is the time constant τ (63.2%) that represents the time needed for the nonstimulated side to be entrained and to reset its frequency.

Normalized differences of frequencies (*ND*_freq_) were computed to better compare the frequency variation between both sides

$${\mathrm{ND}}_{\text{freq}}=|\frac{F_{2}-F_{1}}{\frac{F_{2}+F_{1}}{2}}|$$

For the “triggered” slow oscillations at the side where the frequency was induced (L*_t_*-side), *F*_1_ and *F*_2_ were determined by the local application of glutamate periodicity. For the modulated slow oscillations (R-side), *F*_1_ is the mean obtained in the 90 s previous to the frequency change, and *F*_2_ is the asymptotic value of the exponential.

### Model

#### Ephaptic interaction

To quantify the nonsynaptic interaction between two sides of a sectioned slice, we first estimated the magnitude of EF vector (gradient of electric potential). To this end, we calculated the average extracellular potential associated with a network event triggered by glutamate stimulation in the simulated side of the slice. Under the assumption that the EF originated from the network event and was passively propagated across the cut to the opposite side of the slices, we could estimate the magnitude of related EF vector from the gradient of the potential in the radial direction (across depth) at each lateral position. From this estimate, we took the peak-to-peak amplitude obtaining the magnitude of EF vector as a function of distance from the source.

To estimate the effect of the EF on the membrane depolarization, we used the theory of linear cable in a polarized extracellular medium (*31*). The theory predicts that the membrane becomes polarized by the nonhomogeneous electric potential around it. It also allows us to estimate the magnitude of this depolarization with respect to the spatial frequency and amplitude of the EF. We considered the solution to the cable equation in polarized medium presented by Anastassiou *et al.* (*33*). The membrane potential across a dendrite *V_m_* placed in a nonhomogeneous electric potential is equal to

$$\begin{array}{c} V_{m}(X)=-\frac{\Omega^{2}}{\Omega^{2}+1}\text{sin}(\Omega X+\phi_{S})+\frac{\Omega^{2}}{\Omega^{2}+1}(\frac{\text{cosh}\;(X)}{\text{tanh}\;(L)}\text{cos}\;(\phi_{s})-\\ \frac{\text{cosh}\;(X)}{\text{sinh}\;(L)}\text{cos}(\Omega L+\phi_{s})-\text{sinh}\;(X)\;\text{cos}\;(\phi_{s})) \end{array}$$ (1)

where *L* is the length of the cable.

The solution is given in terms of the dimensionless quantities defined as

$$\Omega =2\pi f_{S}\lambda_{\mathrm{el}}\;X=\frac{x}{\lambda_{\mathrm{el}}}$$

The extracellular potential is given in terms of harmonic functions

$$v_{e}=v_{0}\text{sin}(\;\Omega X+\phi_{S})$$ (2)

and hence the EF is

$$E=-\frac{{dv}_{e}}{\mathrm{dx}}=-E_{0}\text{cos}(\Omega X+\phi_{S})$$ (3)

where *E*_0_ = *v*_0_Ω/λ*_el_*

To obtain a quasi-linear drop of the extracellular potential, we chose low spatial frequencies Ω = 0.001 and ϕ*_s_* = 0. The parameters of the cable were adjusted to standard electrical properties of dendritic trunk with the electrotonic constant of λ*_el_*= 0.76 mm and length *L* = 2 in the units of electrotonic constants. Furthermore, we assume a passive dendrite without active sodium or potassium channels, whereas all the spike-generating currents are located in the soma at the end of the dendrite *X*_soma_ *= L*. The membrane depolarization obtained in the soma is the further used to estimate the effect of the EF on the spike rate in the population.

#### Single-neuron model

We modeled a single neuron as a LIF model, which received conductance-based inhibitory and excitatory inputs

$$C\frac{V_{m}}{\mathrm{dt}}=g_{L}(V_{m}-E_{L})+g_{e}(V_{m}-E_{e})+g_{i}(V_{m}-E_{i})$$ (4)

The total excitatory *g_e_*(*t*) and inhibitory *g_i_*(*t*) conductances were modeled as shot noise processes with alpha kernels of rates ν*_e_* and *ν_i_*, time constants τ*_e_*, and τ*_i_*, and amplitudes *G_e_* and *G_i_*. The rates were adjusted such that the excitatory and inhibitory synaptic currents were approximately balanced producing a subthreshold mean membrane potential. Spikes were elicited when the membrane potential *V_m_*(*t*) crossed a specific threshold *V*_θ_ and the *V_m_*(*t*) was reset to fixed potential *V*_reset_ following each spike. In the balanced regime, these threshold crossings were caused by random fluctuations due to the randomness of the input spikes rather than mean depolarization. At time *t* = 100 ms, we injected a depolarizing current of intensity *I* = 25 pA, which produced a depolarization equal to the one predicted from ephaptic interactions. We estimated the mean firing rate of the neuron before and after injection of the current by averaging over *n* = 5000 repetitions of the simulation with random initial conditions (starting with the same steady-state membrane potential). Parameters of the LIF can be found in table S1. Please, notice that the LIF model was used only to investigate how the EF-induced depolarization modeled with the mean field model (see the “Population model” section next) affected the firing rate; however, there is no integration of both models.

#### Population model

The membrane depolarization induced by the extracellular field is amplified by the recurrent network. To model this phenomenon, we adapted a simplified mean-field population model of the network activity (*49*). In the first order of approximation, the population dynamics can be described by the mean firing rates of excitatory (Eq. 5) and inhibitory (Eq. 6) populations

$$\tau_{e}\frac{v_{e}}{\mathrm{dt}}=-v_{e}+n_{e}f_{e}(v_{e}+v_{\text{ext}},v_{i})+\sigma_{e}\eta_{t}$$ (5)

$$\tau_{i}\frac{v_{i}}{\mathrm{dt}}=-v_{i}+n_{i}f_{i}(v_{e}+v_{\text{ext}},v_{i})+\sigma_{i}\eta_{t}$$ (6)

where *f_e_* and *f_i_* are the transfer functions for inhibitory and excitatory neurons, respectively, and *n_e_* and *n_i_* are the sizes of excitatory and inhibitory populations (*n_e_*/*n_i_* = 4). In addition to the recurrent inputs from the inhibitory *n_i_* and excitatory *n_e_* population, excitatory neurons also receive external excitatory inputs *v*_ext_. η*_t_* denotes a sample of a noncorrelated standard Gaussian noise (white noise), σ*_i_* and σ*_e_* are the SD of the noise for excitation, while there was no added noise for inhibition. These equations were solved numerically using stochastic Euler method, such that the SD of the (discrete) Gaussian noise was scaled with the square root of the integration time step Δ*_t_*.

We use the form of transfer functions suggested by Kuhn *et al.* (*50*). In brief, this model approximates the firing rate of a neuron by a nonlinear function of the membrane fluctuation statistics: mean membrane potential μ*_U_*, SD of membrane potential σ*_U_*, and effective membrane time constant τ_eff_. In the conductance-based model with alpha synapses they are equal to

$$\langle g_{\text{tot}}\rangle =g_{l}+\langle g_{e}\rangle +\langle g_{i}\rangle =g_{l}+\sum_{s\in \{e,i\}}v_{s}B_{s}T_{s}$$ (7)

$$\mu_{U}=\frac{E_{l}g_{l}+E_{e}\langle g_{e}\rangle +E_{i}\langle g_{i}\rangle }{\langle g_{\text{tot}}\rangle }$$ (8)

$$\tau_{\text{eff}}=C/\langle g_{\text{tot}}\rangle$$ (9)

$$\sigma_{U}=\sum_{s\in \{e,i\}}v_{s}(\tau_{\text{eff}}+T_{S}){\left[\frac{(E_{S}-\mu_{U})B_{s}T_{s}\tau_{\text{eff}}}{2C(\tau_{\text{eff}}+T_{s})}\right]}^{2}$$ (10)

Given these statistics derived analytically, the firing rate can be phenomenologically described by the following nonlinear relation

$$f(v_{e},v_{i};\theta )=\frac{1}{\tau_{\text{eff}}(v_{e},v_{i})}\text{erfc}[\frac{\theta -\mu_{U}(v_{e},v_{i})}{\sqrt{2\sigma_{U}}(v_{e},v_{i})}]$$ (11)

For inhibitory neurons, we decrease the threshold for spiking such that their firing rate is higher than that of the excitatory population, such that *f_e_*(*v_e_*, *v_i_*) = *f* (*v_e_*, *v_i_*; θ*_e_*) and *f_i_*(*v_e_*, *v_i_*) = *f* (*v*_*e*,_ *v_i_*; θ*_i_*). We found that under physiological values of the parameters, the model manifests two stable states: the Up state and the Down state. To obtain the oscillations between these two states, we introduced the adaptation in the excitatory population, which is governed by the following dynamic equation

$$\tau_{\text{adapt}}\frac{{\mathrm{dU}}_{\text{adapt}}}{\mathrm{dt}}=-U_{\text{adapt}}+\beta v_{\text{exc}}$$ (12)

The adaptation parameter β and the SD of noise σ_exc_ were adjusted to match the frequency of the Up state observed in the experiments. The adaptation potential *U*_adapt_ is subtracted from the mean membrane potential μ*_U_* of the excitatory population. We implemented two mean-field models of excitatory and inhibitory populations and coupled them by means of the EF through electric dipole interactions.

Specifically, we modified the membrane potential of the excitatory and inhibitory function as a function of the population rate of the excitatory neurons. In particular, the modified mean membrane potential of the L-side population was defined as

$${\bar{\mu }}_{U}^{L}=\mu_{U}+\gamma_{\text{ephaptic}}v_{\text{exc}}^{R}$$ (13)

where the ephaptic coupling coefficient γ_ephaptic_ was estimated from the mean membrane depolarization caused by the ephaptic interaction Δ*V*_ephaptic_ divided by the mean excitatory population rate in an Up state of an uncoupled model 〈*V*_exc_〉*_UP_*. The values of the parameters of this mean-field model can be found in table S2.

#### Phase locking index

In the model, we quantified the strength of modulation by so-called PLI (*51*), which measures the similarity of oscillatory phases, in other words, the time invariance of one location versus another one with respect to their oscillatory cycle (fig. S6). First, we low-pass–filtered the firing rates of excitatory population at both sides (corner frequency, 5 Hz; IIR filter; order 17). From these band-limited traces, we estimated the instantaneous phases of the Up/Down oscillation on the L-side ϕ_L_(*t_i_*) and R-side ϕ_R_(*t*) using the Hilbert transform. Last, we calculated the difference between these phases and quantified its spread by means of the mean vector length

$$\text{PLI}=|\frac{1}{N}\sum_{i=0}^{N}\text{exp}\{-i[\phi_{L}(t_{i})-\phi_{R}(t_{i})]\}|$$ (14)

The PLI has a value in the range from 0 to 1, where 0 corresponds to independent oscillations and 1 to perfect phase synchronization. Its significance was assessed by computing the SE of the PLI.

## SUPPLEMENTARY MATERIALS

Supplementary material for this article is available at http://advances.sciencemag.org/cgi/content/full/7/10/eabc7772/DC1

## Appendix

View/request a protocol for this paper from *Bio-protocol*.
